# Investigation of Inclusion States of Silicate and Carbonate Ions in Hydroxyapatite Particles Prepared under the Presence of Sodium Silicate

**DOI:** 10.3390/biomimetics7020040

**Published:** 2022-04-01

**Authors:** Tania Guadalupe Peñaflor Galindo, Kazuto Sugimoto, Shota Yamada, Taito Sugibuchi, Zizhen Liu, Motohiro Tagaya

**Affiliations:** 1 Department of General Education, National Institute of Technology, Nagaoka College, 888 Nishikatakai, Nagaoka 940-8532, Niigata, Japan; taniap@nagaoka-ct.ac.jp; 2 Department of Materials Science and Technology, Nagaoka University of Technology, Kamitomioka 1603-1, Nagaoka 940-2188, Niigata, Japan; k_sugimoto@stn.nagaokaut.ac.jp (K.S.); shota_yamada@mst.nagaokaut.ac.jp (S.Y.); sugibuchi0315@gmail.com (T.S.); zizhen_liu@stn.nagaokaut.ac.jp (Z.L.)

**Keywords:** hydroxyapatite, nanoparticles, silicate ion reaction, oligomeric silicates, solid-state ^29^Si–NMR

## Abstract

Biological hydroxyapatite (HA) contains the different minor ions which favour its bio-reactivity in vivo. In this study, the preparation of HA particles containing both silicate and carbonate ions under the presence of sodium silicate was investigated, and the physicochemical properties were evaluated according to the contents and states of silicate and carbonate ions. The increment in the silicate ion reduced the crystallinity and expanded the crystalline size along with *a*-axis. Solid-state ^29^Si–NMR spectra indicated the increase in the adsorption of oligomeric silicate species on the HA particle surfaces in addition to the substitution state of silicate ions, suggesting the occurrence of the surface coating of silicates on the surfaces. The possible states of carbonate and silicate ions at the HA surfaces will provide the bioactivity.

## 1. Introduction

The similarity in composition and structure of hydroxyapatite (HA, Ca_10_(PO_4_)_6_(OH)_2_) to the inorganic phase of bones and teeth and its biocompatibility allow it to be used for biomedical applications [1,2]. The low-crystalline HA is suitable for ion substitution in both calcium (Ca^2+^) and phosphate (PO_4_^3−^) ions due to its labile crystal structure [3]. The biological HA usually contains the different minor ions such as Na^+^, K^+^, Mg^2+^, Sr^2+^, F^−^, Cl^−^, carbonate (CO_3_^2−^), and silicate (SiO_4_^4−^) ions [4,5,6]. The substitution or inclusion of these minor ions in HA changes its morphology, crystalline structure and physicochemical properties [7], leading to the enhanced bioactivity [8,9]. Among them, the important ions in the synthetic HA for biological applications are the SiO_4_^4−^ and CO_3_^2−^ ions since the mineral bone contains substantial amounts of the ions [10]. In biological bones, the contents of CO_3_^2−^ and SiO_4_^4−^ ions are approximately 4–8 wt% [8,11] and 0.4–0.5 wt% [12,13], respectively. The SiO_4_^4−^ ions (e.g., silicate ion) are indispensable in the early stages of bone and cartilage formation, since they promote the mineralization, contributing to proper osteointegration and helping to bond the implanted material with the biological bone [9,14]. Thus, the incorporation of silicate ions in the HA structure can significantly improve the biological reactivity of HA [15]. On the other hand, the PO_4_^3−^ ions of HA can be substituted by the CO_3_^2−^ ions, generating the B–type carbonated hydroxyapatite (CHA) [16,17]. The B–type CHA has greater solubility, subserving the Ca^2+^ and PO_4_^3−^ ion concentration, which favours the osteointegration [18,19]. Furthermore, CHA enhances the osteoclast resorption allowing new bone formation [20,21]. Therefore, the understanding of the inclusion of silicate and carbonate ions in HA is the basis for effective control of the bioactivity. Although diverse studies have been carried out on the substitution of carbonate and silicate ions in HA [22], the mechanism of substitution and/or inclusion in low-crystalline HA has not been completely clarified [23,24,25].

The aim of this study is to control the simultaneous incorporation of SiO_4_^4−^ and CO_3_^2−^ ions into HA to obtain low-crystalline HA particles (SiHA) under the presence of sodium silicate in order to increase the HA bioreactivity. The preparation conditions are similar to the physiological conditions used to obtain the particles that are close to the biological HA. The mechanism of substitution with SiO_4_^4−^ and CO_3_^2−^ ions in the HA particles was investigated using the Fourier transform infrared spectrometer, X-ray diffraction, field emission scanning electron microscope, and solid-state ^29^Si–NMR spectra recorded by dipolar decoupled magic-angle spinning.

## 2. Materials and Methods

The SiHA particles with the assumed formula of Ca_10_(PO_4_)_6−*x*_(SiO_4_)*_x_*(OH)_2−*x*_ (*x* = 0~8.0, Ca/(P + Si) = 1.67) were synthesized. Dipotassium hydrogen phosphate (K_2_HPO_4_, FUJIFILM Wako Pure Chemical Co. (Osaka, Japan), purity: 99.0+ wt%), sodium silicate (Water glass, Na_2_O⋅2SiO_2_ with molar ratio (SiO_2_/Na_2_O): 2.06–2.31, FUJIFILM Wako Pure Chemical Co. (Osaka, Japan)), and calcium chloride dihydrate (CaCl_2_⋅2H_2_O, FUJIFILM Wako Pure Chemical Co. (Osaka, Japan), purity: 99.0–103.0 wt%) were used as the starting reagents. The theoretical silicon concentration in SiHA was set at 0, 0.8, 1.5, 4.0, 6.0 and 8.0 wt%. The detailed initial addition amounts were listed in ESM, Table S1. The required amount of K_2_HPO_4_ and Na_2_O⋅2SiO_2_ were dissolved in deionized water (0.04 L) at 60 °C. Then, the deionized water (0.02 L) containing CaCl_2_⋅2H_2_O (0.01 mol) was added to the phosphate and silicate ion solution with continuous stirring at 60 °C. Then, the pH level was adjusted to be 13, and the solution was stirred at 80 °C. After centrifugation, the solid product was washed with ultrapure water and dried at 65 °C for 24 h, and then heated at 250 °C for 3 h. The samples were named as HA, **0.8**SiHA, **1.5**SiHA, **4.0**SiHA, **6.0**SiHA and **8.0**SiHA depending on the feed Si concentration.

The characterization was performed by using Fourier transform infrared (FT-IR) spectrometer, wavelength dispersive X-ray fluorescence (XRF) spectrometer, X-ray diffraction (XRD), field emission scanning electron microscope (FE-SEM), and solid-state ^29^Si–nuclear magnetic resonance recorded by dipolar decoupled magic-angle spinning (^29^Si–NMR DD-MAS). Based on the FT-IR spectra methodology, the quantification of CO_3_^2−^ ions (wt%) was performed by the relative absorbance area ratio of *v*_1_CO_3_^2−^ (1570–1330 cm^−1^) to *v*_1_*v*_3_PO_4_^3−^ (~900–1230 cm^−1^) [26]. According to Scherer’s equation (*K* = 0.89), crystalline size along the *a*–axis (*D*_300_) was calculated from the half width of the *D*_300_ diffraction patterns in the XRD. In the solid state ^29^Si–NMR spectra, the chemical shift was adjusted by referencing the peak position of tetramethylsilane, and was evaluated by the spectral separation technique based on the peaks of *Q*_0_ (–72 ± 2 ppm) due to isolated silicon unit, *Q*_1_ (–74 ± 2 ppm) due to one Si–O–Si and three Si–OH bonds [27] and *Q*_2_ (–90 ± 2 ppm) due to two Si–O–Si and two Si–OH bonds [28].

## 3. Results and Discussion

**Figure 1:**
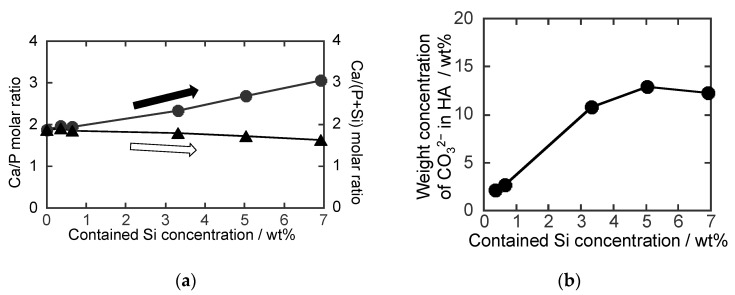
(**a**) Resultant molar ratios of the (●) Ca to P (Ca/P) and the (▲) Ca to (P + Si) (Ca/(P + Si)) and (**b**) carbonate weight concentration in the SiHA particles. Here, the contained Si concentrations were 0.35, 0.65, 3.32. 5.03 and 6.92 wt% for **0.8**SiHA, **1.5**SiHA, **4.0**SiHA, **6.0**SiHA and **8.0**SiHA.

The Si concentrations obtained by XRF were 0.4, 0.7, 3.3. 5.0 and 6.9 wt% for **0.8**SiHA, **1.5**SiHA, **4.0**SiHA, **6.0**SiHA and **8.0**SiHA. As shown in Figure 1a, the Ca/P molar ratio in the SiHA particles increased with the increase in feed Si concentration, suggesting the substitutions of PO_4_^3−^ with SiO_4_^4−^ ions in the HA. Due to the ion substitution, the amount of PO_4_^3−^ ions decreased, causing an increase in the Ca/P molar ratio [18]. On the other hand, the Ca/(Si + P) molar ratio only decreased slightly with the increase in the feeding of Si, indicating the inclusion of SiO_4_^4−^ ions in the HA.

The FT–IR spectra of the SiHA particles (Figure S1) show the stretching vibration band of *v*_1_(PO_4_^3−^) ions at 960 cm^−1^. The band assigned to Si-OH vibrational modes of SiO_4_^4−^ ions appears at 890 cm^−1^. The P–O stretching vibration bands of PO_4_^3−^ ions (1100, 1045 cm^−1^) and the Si–O–Si asymmetric bands of SiO_4_^4−^ ions (1040–1200 cm^−1^) are located very close, which makes interpretation difficult. The bands at around 1470, 1550 cm^−1^ correspond to the asymmetric stretching mode of *v*_3_CO_3_^2−^ ions. The band at ~3570 cm^−1^ is assigned to the OH^−^ stretching mode [29,30,31,32]. It was observed that the increase in Si concentration caused an increase in the intensity of the Si–OH band and a decrease in the intensity of the *v*_1_(PO_4_^3−^) band, corroborating the inclusion of SiO_4_^4−^ ions by the substitution of PO_4_^3−^ ions. Furthermore, increasing the Si concentration increased the intensity of the CO_3_^2−^ bands, indicating the simultaneous substitution of PO_4_^3−^ ions with SiO_4_^4−^ and CO_3_^2−^ ions in the HA to form SiHA. This can be described by the possible formula (e.g., 2PO_4_^3−^→SiO_4_^4−^ + CO_3_^2−^) [33]. The incorporation of SiO_4_^4−^ ions caused a decrease in the intensity of the OH^−^ band until it almost disappeared in **8.0**SiHA, implying the loss of OH^−^ ions to compensate for the excess negative charges generated by the substitution of the PO_4_^3−^ ions with more negative ions (SiO_4_^4−^). Using the Kröger–Vink notation [34], the charge compensation mechanism [35] can be expressed as follows:

(1) $${2\mathrm{SiO}}_{2}\;{+\;2\mathrm{PO}}_{4}^{x}{}_{{\mathrm{PO}}_{4}}{\;+\;2\mathrm{OH}}_{\mathrm{OH}}^{x}\;\to {\;2\mathrm{SiO}}_{4_{{\mathrm{PO}}_{4}}}^{\;,}{\;+\;2V}_{\mathrm{OH}\;}^{\;\cdot }{\;+\;P}_{2}O_{5}{\;+\;H}_{2}O$$

Therefore, the increase in Si concentration effectively caused an increase in the content of CO_3_^2−^ ions, as shown in Figure 1b, indicating that the presence of SiO_4_^4−^ ions induced the inclusion of CO_3_^2−^ ions in HA. The incorporation of SiO_4_^4−^ and CO_3_^2−^ ions into HA increased the defects in the HA structure, resulting in more vacancies of OH^−^ ($V_{\mathrm{OH}}^{\cdot }$) due to more substitutions of PO_4_^3−^ ions. The simultaneous substitution of the PO_4_^3−^ ions in the HA with SiO_4_^4−^ and CO_3_^2−^ ions can be described as follows [13,36]

(2) $${2\mathrm{SiO}}_{2}{\;+\;2\mathrm{CO}}_{2}{\;+\;4\mathrm{PO}}_{4}^{x}{}_{{\mathrm{PO}}_{4}}{\;+\;2\mathrm{OH}}_{\mathrm{OH}}^{x}{\;+\;2H}_{H}^{x}\;\to {\;2\mathrm{SiO}}_{4_{{\mathrm{PO}}_{4}}}^{\;,}{\;+\;2V}_{\mathrm{OH}}^{\;\cdot }{\;+\;2\mathrm{CO}}_{3_{{\mathrm{PO}}_{4}}}^{\;\cdot }{\;+\;2V}_{H}^{\;,}{\;+\;2P}_{2}O_{5}{\;+\;2H}_{2}O$$

**Figure 2:**
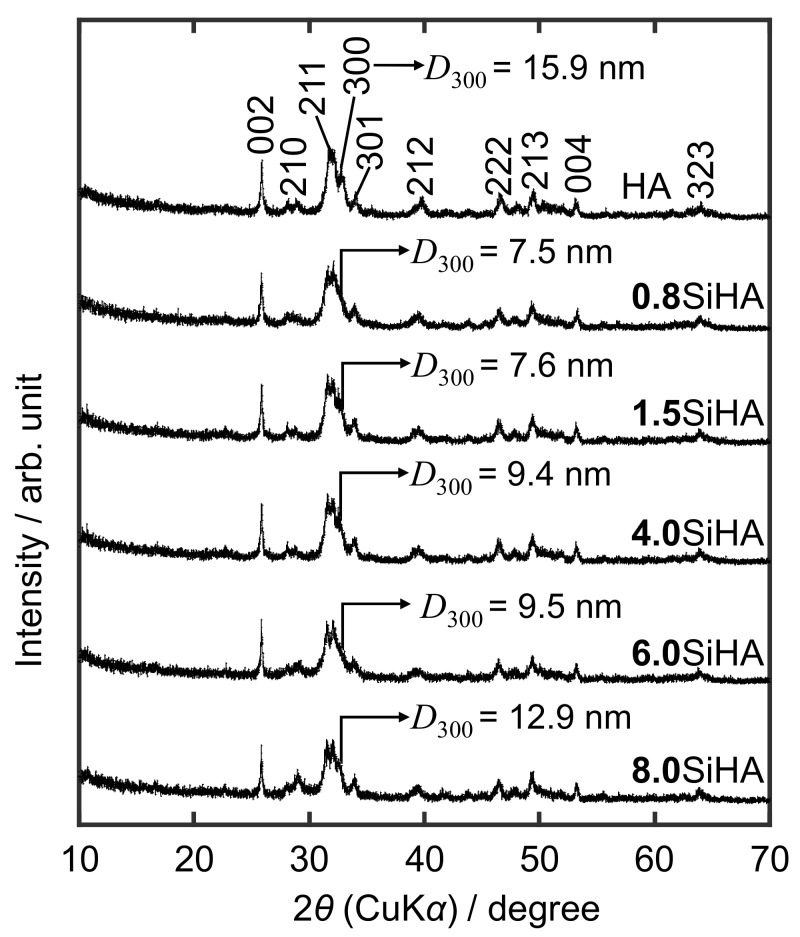
XRD patterns of the HA and SiHA particles (Inset: *D*_300_ values).

Figure 2 shows the XRD patterns of HA and SiHA particles at the different Si concentrations. All the patterns were ascribed to a HA (Ca_10_(PO_4_)_6_(OH)_2_, ICDD: 00-009-0432). It was observed that the increase in Si concentration resulted in a decrease in crystallinity, which was caused by the defects and the formation of $V_{OH}^{\cdot }$ due to the substitution of PO_4_^3−^ ions with SiO_4_^4−^ and CO_3_^2−^ ions in the HA structure. The calculated *D*_300_ crystalline sizes along with the *a*–axis of all the SiHA samples were smaller than the HA (Figure 2 (inset)) due to the incorporation of SiO_4_^4−^ and CO_3_^2−^ ions into the HA structure [37]. It was observed that the value of *D*_300_ first decreased (**0.8**SiHA) and then began to increase with increasing the Si concentration. Especially, **8.0**SiHA had the highest value of *D*_300_, suggesting that the substitution of PO_4_^3−^ ions with SiO_4_^4−^ ions would occur predominantly, since the radius of the Si^4+^ (0.042 nm) is greater than that of P^5+^ (0.035 nm), and the bond length of the Si–O bond (0.16 nm) is greater than that of the P–O bond (0.15 nm) [20,38], which must result in a higher *D*_300_ value. These results are consistent with the crystal sizes observed in FE-SEM images (Figure S2). In the images, all the SiHA particles exhibit needle-like shapes with the smaller sizes than HA, and theaggregation forms were observed.

**Figure 3:**
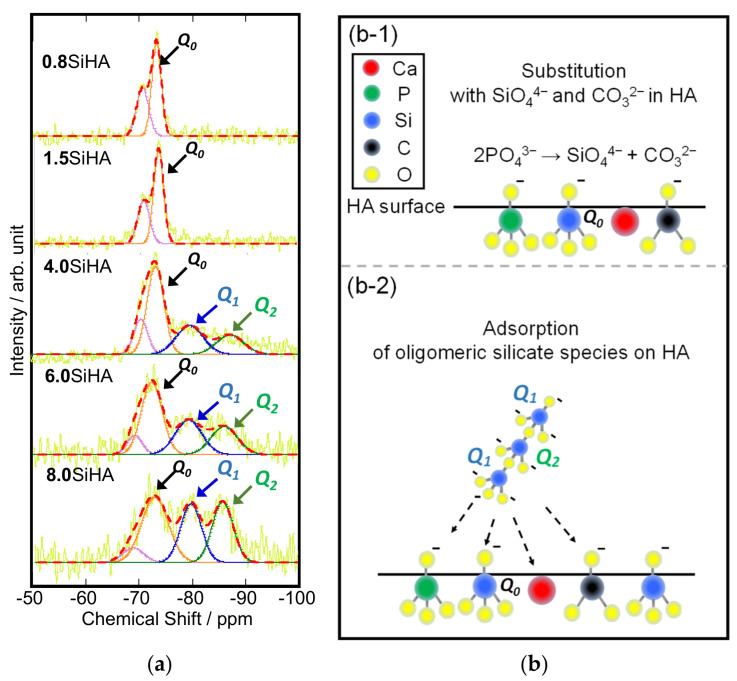
(**a**) Deconvolution curves (red dotted lines) and fitting results (coloured lines) of solid-state ^29^Si–NMR DD-MAS spectra of the SiHA particles with the *Q_n_* (*n* = 0, 1, 2) derived from raw data (yellow lines). Schemes of the possible states of carbonate and silicate ions at the HA surfaces for the case in (**b-1**) **0.8**SiHA and **1.5**SiHA and (**b-2**) **4.0**SiHA, **6.0**SiHA and **8.0**SiHA. The increment in the Si concentration increases the relative *Q*_2_ peak intensity, suggesting the absorption of oligomer derived from unsubstituted silicate ions on the HA surface.

Figure 3a shows the deconvolution curves and fitting results of the spectra of the SiHA particles provided by their *Q*_0_~*Q*_2_ components. At the lower Si concentration (**0.8**SiHA and **1.5**SiHA), only *Q*_0_ peak was observed. However, at the higher Si concentration (**4.0**SiHA, **6.0**SiHA and **8.0**SiHA), the *Q*_1_ and *Q*_2_ peaks appeared. The *Q*_0_ peak of the SiO_4_ tetrahedron corresponded to the stretching Si–O band in the FT–IR spectra of SiHA. The possible inclusion mechanism of the silicates ions into HA structure is shown in Figure 3b. It was suggested that the substitution with SiO_4_^4−^ ions in the HA structures was related to the inclusion of CO_3_^2−^ ions, which was proposed by the reaction mechanism based on the substitution of phosphate ions in Figure 3(b-1). Polymerized silicate chains (e.g., oligomeric silicates) due to *Q*_1_ and *Q*_2_ peaks appeared at the Si concentration of 3.32 wt%, suggesting that the unsubstituted SiO_4_^4−^ ions began to adsorb on the SiHA with the polymerization and condensation reactions. At the Si concentrations of 5.03 and 6.92 wt%, the intensities of *Q*_1_ and *Q*_2_ increased, suggesting that the unsubstituted SiO_4_^4−^ ions started to form oligomeric fragments and adsorbed on the SiHA. These results indicated that the oligomeric fragments are larger in the presence of more unsubstituted SiO_4_^4−^ ions as shown in Figure 3(b-2).

## 4. Conclusions

The inclusion of both silicate and carbonate ions was successfully achieved in the presence of sodium silicate. The content of silicate ions increased with increasing Si feed. The silicate ions promoted the inclusion of carbonate ions in the HA. The substitution of phosphate ions with silicate and carbonate ions into the HA structure produced defects and hydroxyl vacancies, generating a loss in crystallinity and smaller crystalline sizes. The substitution of phosphate ions was mainly dominated with silicate ions in the structure, inducing the particle growth along with the *a*–axis. The results of solid-state ^29^Si–NMR DD-MAS demonstrated that at the lower Si feed, only ion substitution has occurred. At the higher Si content, the remaining silicate ions with the saturation of substitution formed the (poly)silicate species (i.e., oligomeric silicates). It was observed that these (poly)silicate species have a strong tendency to adsorb to the HA surfaces, modulating the crystal growth of the SiHA. Thus, the solid state ^29^Si-NMR spectra indicated that upon reaching saturation in the substitution of phosphate ions with silicate ions, the remaining silicate ions polymerized to form (poly)silicate species adsorbed on the surface of the HA, suggesting the appearance of the silicate surface layer on the SiHA surfaces. There is a possibility to control the silicate ion states, the inclusion amount of carbonate ions in SiHA and its crystalline size by the feed concentration of sodium silicate. The incorporation of silicate and carbonate ions into the HA structure is expected to enhance the bioactivity of the SiHA particles.

## Data Availability

Data available on request from the authors.

## Supplementary Materials

The following supporting information can be downloaded at: https://www.mdpi.com/article/10.3390/biomimetics7020040/s1, Table S1: Added amounts of the reagents in the synthesis of the SiHA particles; Figure S1: FT–IR spectra of the HA and SiHA particles; Figure S2: FE−SEM images of the HA and SiHA particles.
