# Supplementary material for: Investigation of Inclusion States of Silicate and Carbonate Ions in Hydroxyapatite Particles Prepared under the Presence of Sodium Silicate
Source: Biomimetics (Basel). 2022 Apr 1;7(2):40. doi: 10.3390/biomimetics7020040 (PMC9036305; doi:10.3390/biomimetics7020040)
Supplement: Supplementary file 1 [file biomimetics-07-00040-s001.zip › biomimetics-1664301-supplementary.pdf]

Supplementary Materials

Investigation of inclusion states of silicate and carbonate ions in hydroxyapatite particles prepared under the presence of sodium silicate

Tania Guadalupe Peñaflor Galindo, [a] Kazuto Sugimoto, [b] Shota Yamada, [b] Taito Sugibuchi, [b] Zizhen Liu, [b] Motohiro Tagaya [b,*]

[a] *Department of General Education, National Institute of Technology, Nagaoka College, 888 Nishikatakai, Nagaoka, Niigata 940-8532, Japan*

[b] *Department of Materials Science and Technology, Nagaoka University of Technology, Kamitomioka 1603-1, Nagaoka, Niigata 940-2188, Japan*

*** Author to whom correspondence should be addressed:**

Tel: +81-258-47-9345, Fax: +81-258-47-9300, E-mail: tagaya@mst.nagaokaut.ac.jp

Table S1

Table S1. Added amounts of the reagents in the synthesis of the SiHA particles. Here, the samples were named as HA, 0.8Si HA, 1.5Si HA, 4.0Si HA, 6.0Si HA, 8.0Si HA depending on the inital Si concentration.

Sample name	Si concentration (wt%)	K_2HPO_4 (mmol)	$Na_2O \cdot 2SiO_2$ (mmol)	$CaCl_2 \cdot 2H_2O$ (mmol)	Ca / (P+Si) molar ratio
HA	0.00	6.12	0.000	10.0	1.67
**0.8**SiHA	0.80	5.83	0.253		
**1.5**SiHA	1.50	5.58	0.272		
**4.0**SiHA	4.00	4.70	1.24		
**6.0**SiHA	6.00	4.02	1.83		
**8.0**SiHA	8.00	3.36	2.42		

Figure S1

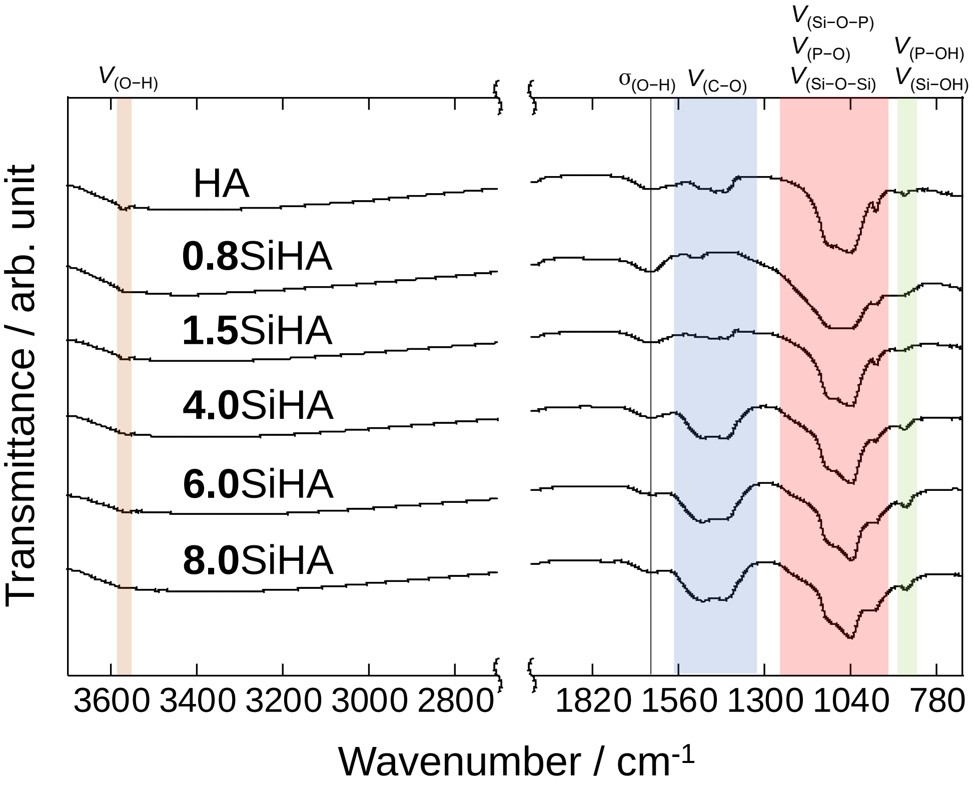

Figure S1. FT–IR spectra of the HA and SiHA particles.

Figure S2

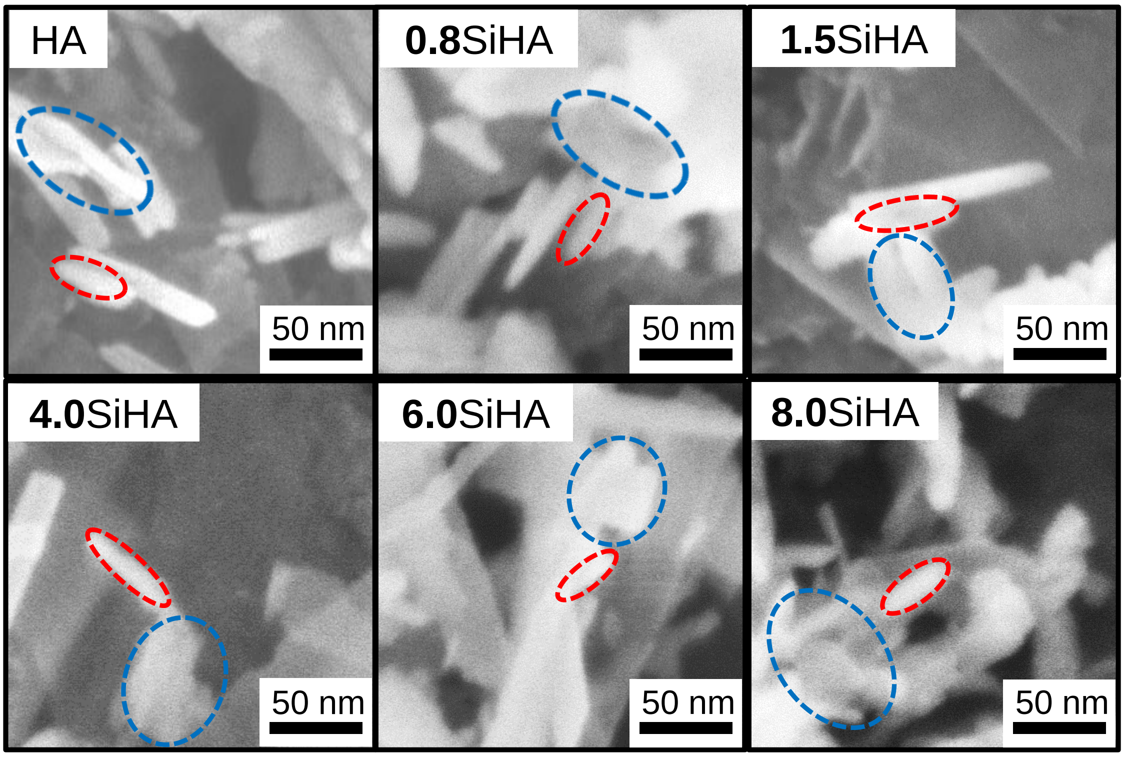

Figure S2. FE−SEM images of the HA and SiHA particles. The red and blue dotted circles indicate the primary and aggregate particles, respectively.
